# Carbon ion radiotherapy for mesonephric adenocarcinoma of the uterine cervix: a case report

**DOI:** 10.1186/s13256-024-04548-6

**Published:** 2024-05-09

**Authors:** Nao Kobayashi, Takahiro Oike, Ken Ando, Kazutoshi Murata, Tomoaki Tamaki, Shin-ei Noda, Kayoko Kogure, Sumihito Nobusawa, Tetsunari Oyama, Tatsuya Ohno

**Affiliations:** 1 Department of Radiation Oncology, Kyorin University, 6-20-2 Shinkawa, Mitaka-Shi, Tokyo 181-8611 Japan; 2 Department of Radiation Oncology, Gunma University Graduate School of Medicine, 3-39-22 Showa-Machi, Maebashi-Shi, Gunma 371-8511 Japan; 3 Gunma University Heavy Ion Medical Center, 3-39-22 Showa-Machi, Maebashi-Shi, Gunma 371-8511 Japan; 4 QST Hospital, National Institutes for Quantum Science and Technology, 4-9-1 Anagawa, Inage-Ku, Chiba-Shi, Chiba 263-8555 Japan; 5 Department of Radiation Oncology, Fukushima Medical University School of Medicine, 1 Hikarigaoka, Fukushima-Shi, Fukushima 960-1295 Japan; 6 Department of Health Risk Communication, Fukushima Medical University School of Medicine, 1 Hikarigaoka, Fukushima-Shi, Fukushima 960-1295 Japan; 7 Department of Radiation Oncology, Saitama Medical University International Medical Center, 1397-1 Yamane, Hidaka-Shi, Saitama 350-1298 Japan; 8 Department of Obstetrics and Gynecology, Isesaki Municipal Hospital, 12-1 Tsunatorihonmachi, Isesaki-Shi, Gunma 372-0817 Japan; 9 Department of Human Pathology, Gunma University Graduate School of Medicine, 3-39-22 Showa-Machi, Maebashi-Shi, Gunma 371-8511 Japan; 10 Department of Diagnostic Pathology, Gunma University Graduate School of Medicine, 3-39-22 Showa-Machi, Maebashi-Shi, Gunma 371-8511 Japan

**Keywords:** Mesonephric adenocarcinoma, Uterine cervical cancer, Carbon ion radiotherapy, Image-guided adaptive brachytherapy, Lung metastases, Case report

## Abstract

**Background:**

Mesonephric adenocarcinoma is an extremely rare subtype of uterine cervical cancer that is associated with a poor prognosis and for which a standardized treatment protocol has not been established. Carbon ion radiotherapy (CIRT) is an emerging radiotherapy modality that has been shown to have a favorable anti-tumor effect, even for tumors resistant to conventional photon radiotherapy or chemotherapy. However, there is no report on CIRT outcomes for mesonephric adenocarcinoma of the uterine cervix.

**Case presentation:**

We treated a 47-year-old Japanese woman with mesonephric adenocarcinoma of the uterine cervix (T2bN0M0 and stage IIB according to the 7th edition of the Union for International Cancer Control and International Federation of Gynecology and Obstetrics, respectively) with CIRT combined with brachytherapy and concurrent chemotherapy. CIRT consisted of whole pelvic irradiation and boost irradiation to the gross tumor; 36.0 Gy (relative biological effectiveness [RBE]) in 12 fractions and 19.2 Gy (RBE) in 4 fractions, respectively, performed once a day, four times per week. Computed tomography-based image-guided adaptive brachytherapy was performed after completion of CIRT, for which the D_90_ (i.e., the dose prescribed to 90% of the target volume) for the high-risk clinical target volume was 20.4 Gy in a total of 3 sessions in 2 weeks. A weekly cisplatin (40 mg/m^2^) dose was administered concomitantly with the radiotherapy for a total of five courses. From 4 months post-CIRT, the patient developed metastasis of the lung, with a total of 10 lung metastases over 70 months; these lesions were treated on each occasion by photon stereotactic body radiotherapy and/or systemic therapy. At 8 years from initial treatment (i.e., 2 years after the last treatment), the patient is alive without any evidence of recurrence and maintains a high quality of life.

**Conclusions:**

This is the first report of CIRT for treatment of mesonephric adenocarcinoma of the uterine cervix. The present case indicates the potential efficacy of CIRT in combination with brachytherapy for treatment of this disease.

## Background

Cervical cancer causes more than 0.3 million deaths worldwide annually [1], and adenocarcinomas comprise approximately 25% of cervical cancers [2]. Mesonephric adenocarcinoma (MNA) is an extremely rare subtype that originates from the remnants of persistent mesonephric ducts and accounts for less than 1% of all cervical adenocarcinomas [3]. Because of its rarity, the biological behavior of MNA and its prognosis are unclear. Nevertheless, previous case reports suggest that the prognosis for MNA is worse than that for cervical cancer of other histological types [4, 5]. The recurrence rate of stage I MNA is approximately 30%, which is prominent even among those adenocarcinomas known to have a worse prognosis than squamous cell carcinoma [6]. This indicates that the standard treatment for cervical cancer, recommended by the National Cancer Comprehensive Network [7], is insufficient to eradicate MNA, highlighting the need to establish a treatment strategy suited to this disease subset. To date, most MNA cases have been treated with surgical resection in combination with adjuvant or neoadjuvant treatment, depending on the disease stage [4, 5, 8], and there are only a limited number of reports of cases treated with radiotherapy (Table 1).

**Table 1:** Summary of the literature reporting mesonephric carcinoma treated with radiotherapy

Case	Ref	Year	Age	Stage	Primary Tx	Adjuvant Tx	RT details	Rec site (time, Tx)	Outcome
1	[15]	1990	46	NA	HRT	RT	NA		NED (10 mo)
2	[16]	1990	55	NA	HRT + BSO	RT	NA		NED (60 mo)
3	[17]	1995	71	IB	HRT + BSO + LA	RT	NA	Abdomen (4 mo, CT)	DOD (8 mo)
4	[17]	1995	73	IB	HRT + BSO	RT	NA		NED (36 mo)
5	[17]	1995	40	IB	HRT + BSO	RT	NA		NED (27 mo)
6	[18]	2001	72	IB	HRT + BSO + LA	RT	NA	Rectovaginal septum (20 mo, CT)	NED (30 mo)
7	[18]	2001	35	IIB	RT		EBRT + BT	Pelvis (26 mo, CT)	DOD (38 mo)
8	[19]	2004	41	IB	HRT + BSO + LA	RT	NA		NED (136 mo)
9	[20]	2006	54	IB	HRT + BSO + LA	RT	EBRT(50.4Gy) + BT(12.7Gy)		NED (37 mo)
10	[21]	2013	48	IB	HRT	CRT	NA		NED (24 mo)
11	[22]	2013	65	IB	HRT + BSO + LA	RT	BT		NED (6 mo)
12	[5]	2016	66	IIB	CRT	HRT + BSO	EBRT(50Gy/25fr.) + cisplatin		NED (24 mo)
13	[23]	2019	67	IIB	HRT + BSO + LA	CRT	NA		NED (12 mo)

*BSO* bilateral salpingo-oophorectomy, *BT* brachytherapy, *CRT* chemoradiotherapy, *CT* chemotherapy, *DOD* dead of disease, *EBRT* external beam radiotherapy, *fr.* Fractions, *HRT* hysterectomy, *LA* lymphadenectomy, *mo* month, *NA* not applicable, *NED* no evidence of disease, *Rec* recurrence, *Ref* reference, *RT* radiotherapy, *Tx* treatment

Carbon ion radiotherapy (CIRT) is an emerging radiotherapy modality that can achieve a dose distribution that is highly conformal to the target [9]. Additionally, CIRT provides biological advantages not observed in proton or photon therapy, attributed to its high linear energy transfer (LET). CIRT induces increased double-stranded DNA structures, leading to irreversible cell damage independently of the cell cycle phase or oxygenation, more so than lower LET irradiation, such as proton and photon therapy [10–14]. In this manner, CIRT shows excellent anti-tumor effects, suggesting its potential as an option for local treatment to eradicate MNA. However, there is no report on CIRT outcomes for MNA. Here, we report the first case of MNA treated with CIRT in combination with brachytherapy.

## Case presentation

**Fig. 1:**
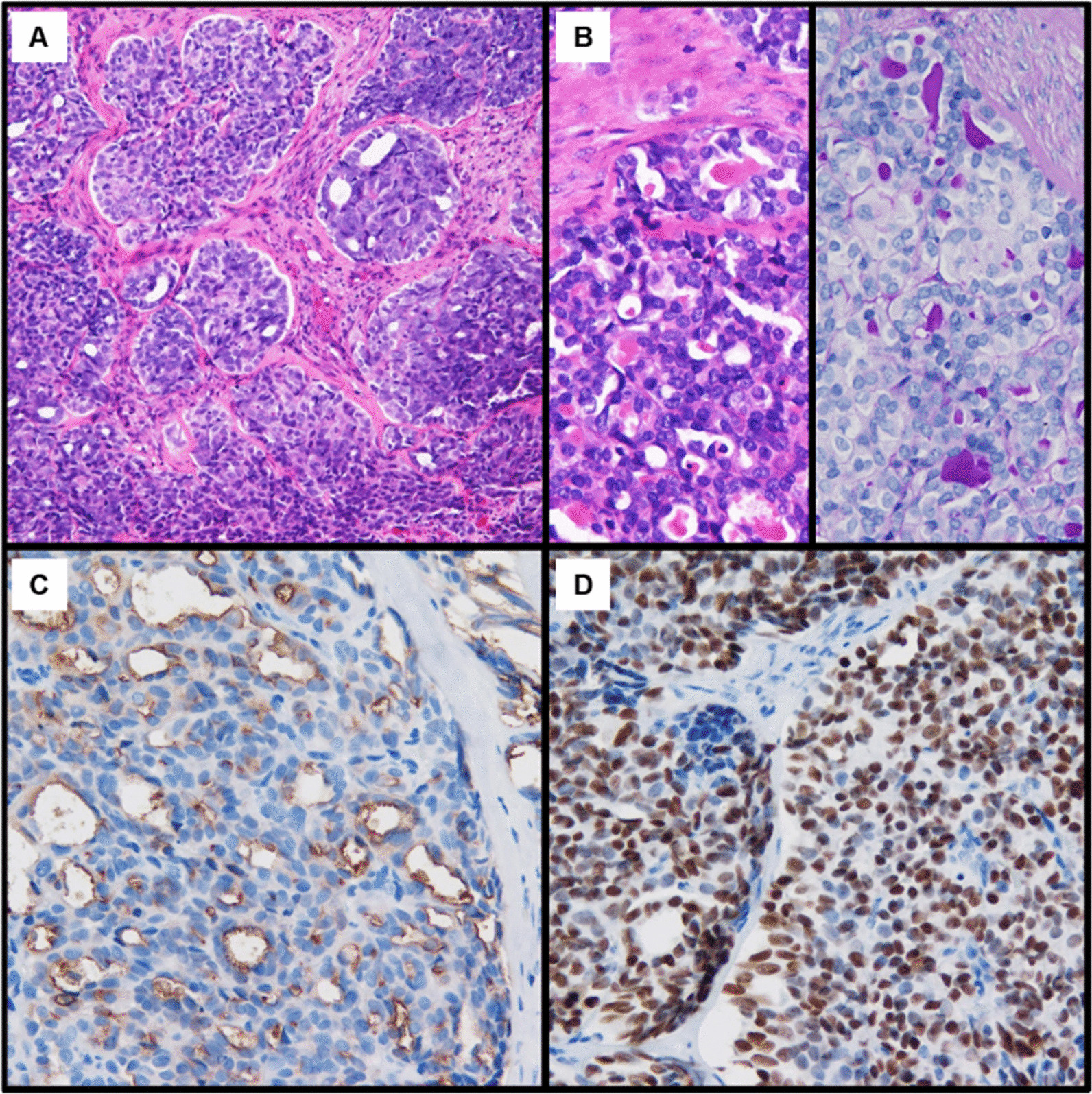
Pathological analysis of the cervical tumor biopsy specimen. **A** Hematoxylin–eosin stained specimen (× 100). **B** Periodic acid-Schiff stained specimen (left panel) and periodic acid-Schiff after diastase digestion stained specimen (right panel) (× 200). **C** Immunohistochemical staining for CD10 (× 200). **D** Immunohistochemical staining for TTF-1 (× 200)

**Fig. 2:**
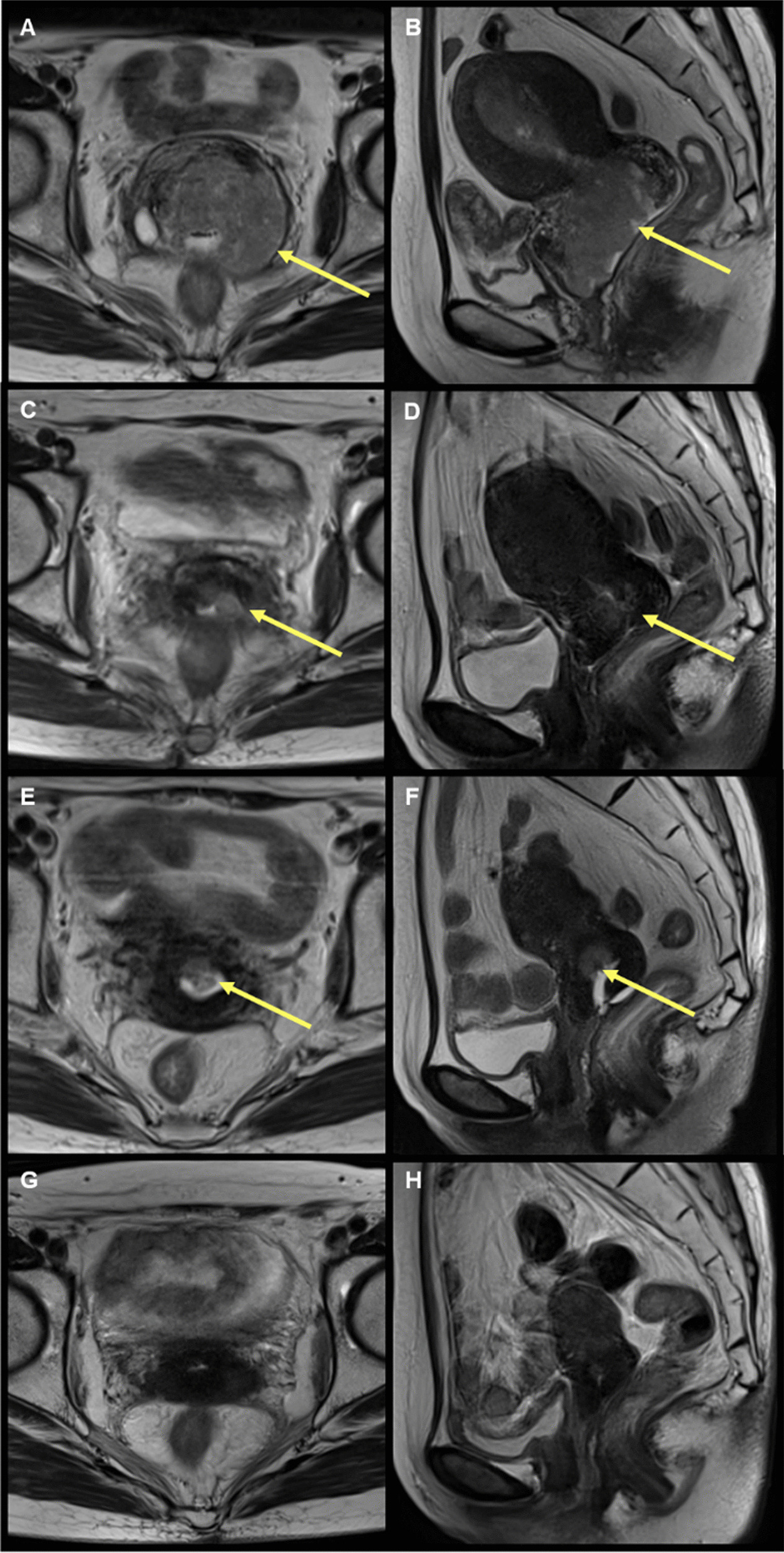
T2-weighted magnetic resonance images of the primary tumor (yellow arrows). Time of diagnosis: axial (**A**) and sagittal (**B**) planes. At 3 months: axial (**C**) and sagittal (**D**) planes. At 6 months: axial (**E**) and sagittal (**F**) planes. At 30 months: axial (**G**) and sagittal (**H**) planes

A 47-year-old Japanese woman was referred to our department of radiation oncology for treatment of locally advanced cervical cancer. The chief complaint was an increased amount of vaginal discharge. Her menstrual cycle was regular with a 40-day interval, and there is no history of irregular vaginal or postcoital bleeding. Additionally, she had no relevant medical history. On histopathological examination of the tumor biopsy specimen, the tumor formed irregular solid sheets and confluent glandular/cribriform structures (Fig. 1A). The glandular structures were lined with flattened to cuboidal or columnar cells, and the lumens occasionally contained periodic acid-Schiff-positive and diastase-resistant eosinophilic secretions (Fig. 1B). On immunohistochemistry, luminal CD10 positivity (Fig. 1C) and diffuse nuclear expression of TTF-1 (Fig. 1D) and PAX8 were observed. The tumor cells were negative for p16, ER and calretinin. These findings led to the diagnosis of MNA. Pelvic examination revealed a cervical mass without vaginal invasion, although with left parametrial involvement that did not reach the pelvic wall. Magnetic resonance imaging (MRI) also showed an irregular tumor (65 mm in diameter) with similar findings to the pelvic examination (Fig. 2A, B). The chest-abdomen-pelvis computed tomography (CT) and 18-fluoro-2-deoxyglucose (F-18-FDG)-positron emission tomography (PET)/CT showed no evidence of metastasis to the lymph nodes or other organs. On the basis of these findings, the disease was staged as T2bN0M0 (based on the 7th edition of the Union for International Cancer Control) and stage IIB (based on the International Federation of Gynecology and Obstetrics 2009).

**Fig. 3:**
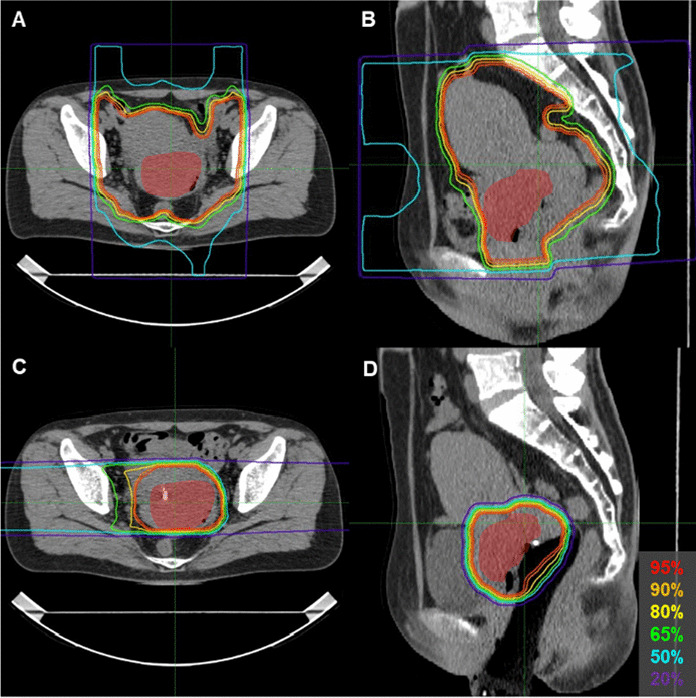
Representative image of the dose distribution of the carbon ion radiotherapy. The gross tumor is depicted in transparent red. Whole pelvic irradiation: axial (**A**) and sagittal (**B**) planes. Local boost irradiation: axial (**C**) and sagittal (**D**) planes

**Fig. 4:**
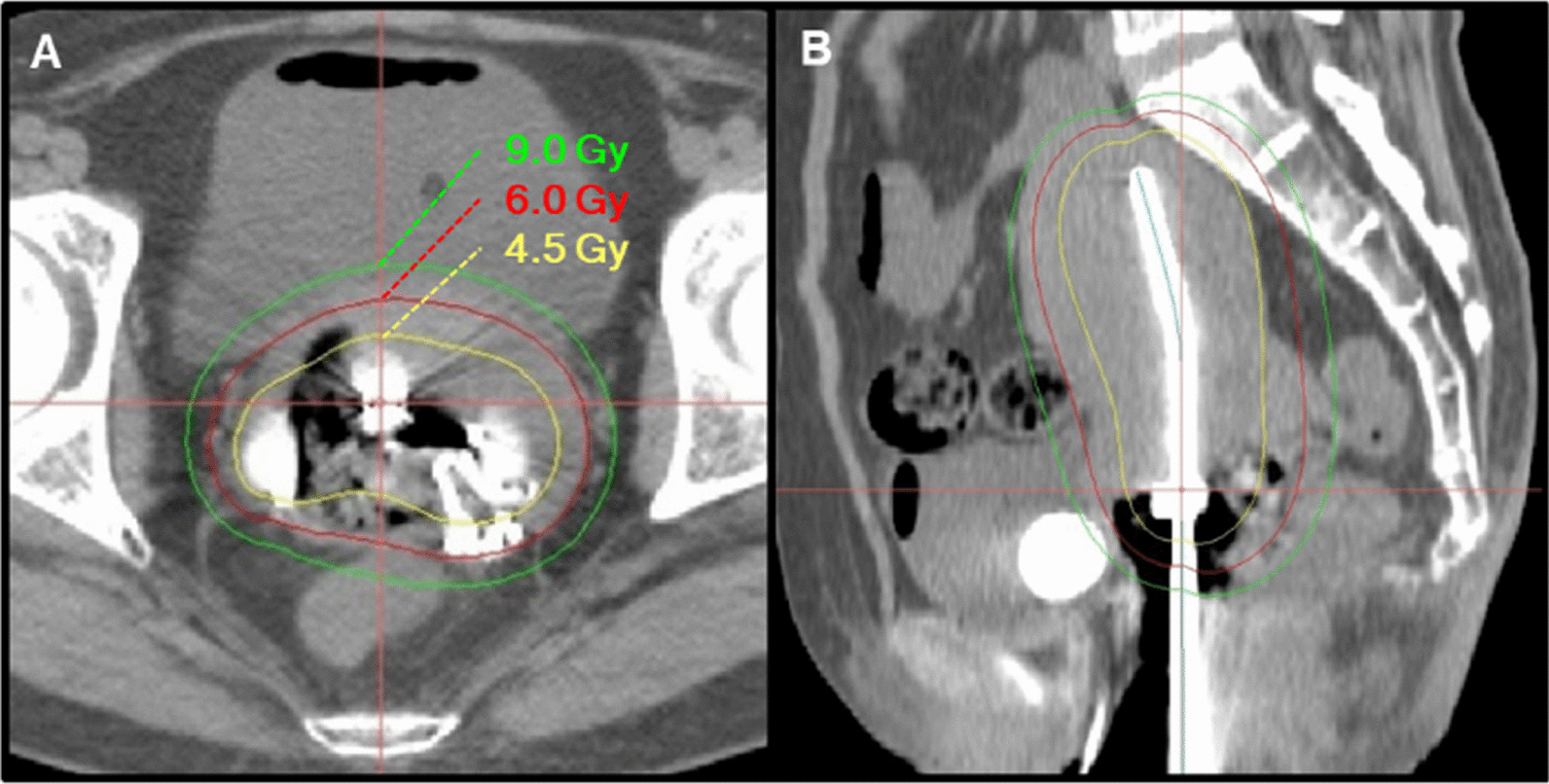
Representative image of the dose distribution for image-guided adaptive brachytherapy using a Fletcher-Suit Asian Pacific applicator. Axial (**A**) and sagittal (**B**) planes

The patient was enrolled in a clinical trial, a prospective interventional study on the safety of carbon ion radiotherapy and image-guided brachytherapy for locally advanced uterine cervical cancer (GUNMA1202), and received definitive treatment consisting of CIRT, ^192^Ir-based high-dose-rate image-guided adaptive brachytherapy (IGABT), and five courses of concurrent weekly cisplatin (40 mg/m^2^). The CIRT consisted of whole pelvic irradiation and local boost irradiation. For the whole pelvic irradiation, a total of 36.0 Gy (relative biological effectiveness [RBE]) in 12 fractions was delivered to the primary tumor site (encompassing the gross tumor, whole uterus, parametria, ovaries, and the upper half of the vagina) and the prophylactic lymph node regions (encompassing the common iliac, internal iliac, external iliac, obturator, and presacral node regions) (Fig. 3A, B). For the local boost irradiation using an inserted vaginal spacer, a total of 19.2 Gy (RBE) in four fractions was delivered to the gross tumor (Fig. 3C, D). CIRT was performed as one fraction per day, four fractions per week. After the local boost irradiation was completed, three sessions of CT-based IGABT using a Fletcher-Suit Asian Pacific applicator (Elekta, Stockholm, Sweden) were performed for a total of 3 sessions in 2 weeks (Fig. 4A, B). The following dose aim for the target, and dose constraints for the organs at risk, were used: D_90_ (i.e., the minimum dose at which 90% of the volume is irradiated) for the high-risk clinical target volume (HR-CTV) greater than 16.5 Gy; and D_2cc_ (i.e., the maximum dose at which 2 cc of the volume is irradiated) of the rectum and sigmoid colon below 16.5 Gy. The resulting HR-CTV D_90_, rectum D_2cc_, and sigmoid colon D_2cc_ were 20.3 Gy, 15.7 Gy, and 14.0 Gy, respectively, showing that the dose aims and constraints were achieved. Five courses of weekly cisplatin at a dose of 40 mg/m^2^ were given during the C-ion RT and brachytherapy period. The first course of cisplatin was administered on day 1 of C-ion RT in principle. Cisplatin was administrated on a different day during the brachytherapy period.

MRI obtained at 3 months (with the first day of treatment defined as Day 1) showed remarkable shrinkage of the cervical tumor to 25 mm in diameter (Fig. 2C, D). The tumor showed further shrinkage to 15 mm in diameter at 6 months (Fig. 2E, F), reaching a radiological complete response at 30 months (Fig. 2G, H). At 9 months, the patient developed rectal bleeding due to radiation proctitis, which was resolved by argon plasma coagulation and hyperbaric oxygen therapy.

In contrast to the control of the primary tumor, the patient developed multiple metastases to the lung (i.e., eight lesions) at 4 months. The patient received six courses of paclitaxel (175 mg/m^2^) plus carboplatin (area under the curve, 5 mg/ml/minute), followed by two sessions of dendritic cell vaccine therapy; these treatments led to a radiological complete response for seven out of the eight lesions, with the other lesion (in the right S1) being stable. The right S1 lesion was treated with photon stereotactic body radiotherapy (SBRT). From 22 to 78 months, the patient received SBRT for a total of 10 metastatic lesions to the lung, i.e., the original right S1 lesion, six new lesions, and three post-chemotherapy relapse lesions (Table 2).

**Table 2:** Summary of stereotactic body radiotherapy for the metastatic tumors in the lung

Treatment #	Months	Location	Tumor diameter	Dose/fr.
1	22	Right S1	8 mm	27 Gy/1 fr.
2	22	Right S4 + S5	5 mm	27 Gy/1 fr.
3	28	Right S3 (behind the sternum)	12 mm	27 Gy/1 fr.
4	34	Right S9	8 mm	27 Gy/1 fr.
5	36	Right S6	8 mm	27 Gy/1 fr.
6	57	Right S3 (mediastinal side)	16 mm	40 Gy/3 fr.
7	62	Left S6	10 mm	45 Gy/3 fr.
8	72	Right S6 + S8	6 mm	52 Gy/4 fr.
9	76	Left S3	5 mm	48 Gy/4 fr.
10	78	Right S3	14 mm	50 Gy/5 fr.

*fr.* fraction(s)

No adverse effects other than asymptomatic pneumonitis with radiological findings only were observed post-SBRT. All lesions treated with SBRT were controlled until the latest follow-up. At 8 years (i.e., 2 years after the last SBRT), the patient is alive without any evidence of recurrence on CT workup every 6 months, and maintains a high quality of life.

## Discussion and conclusions

A systematic review suggests that MNA of the uterine cervix is treated predominantly with surgical resection, and that a common site for recurrence is the abdominal cavity [4]. The remnants of the mesonephric duct are located deep in the parametrium, which can lead to incomplete resection of malignant tumor, resulting in abdominal recurrence, even for early-stage cases [4, 20]. In contrast to surgery, there is little evidence on the efficacy of radiotherapy for MNA of the uterine cervix. To the best of our knowledge, there are only 13 MNA cases treated with radiotherapy reported in the literature (Table 1). Furthermore, in most cases radiotherapy was used in an adjuvant or neoadjuvant setting in combination with radical surgery, making it difficult to estimate the efficacy of radiotherapy for tumor control. There is only one case described in which radiotherapy was used as the primary treatment; this case (i.e., Case 7) was a stage IIB patient who received a combination of photon external beam radiotherapy (EBRT, the prescribed dose was not described) and intracavitary brachytherapy. This patient experienced pelvic recurrence at 26 months, indicating that this treatment was insufficient to achieve pelvic control of the MNA. EBRT for cervical cancers targets the whole pelvis; thus the pelvic recurrence in this case may indicate the radioresistant nature of MNA. Although the majority of cervical cancers are caused by high-risk human papillomavirus (HPV) infection, approximately 15% of cervical adenocarcinomas are unrelated to HPV infection [2], and MNA is classified as a non-HPV-associated adenocarcinoma, which also suggests radioresistance [24, 25].

In contrast to Case 7, the combination of CIRT (55.2 Gy in 16 fractions) and IGABT used in our case of locally advanced MNA achieved pelvic control for 8 years. Pre-clinical studies suggest that carbon ions induce huge and complex DNA double-strand breaks [26], which lead to efficient induction of cell death in photon-resistant cancer cells through a process called mitotic catastrophe [27, 28]. HPV-negative squamous cell carcinoma of the head and neck has been suggested to be resistant to photon radiation compared to HPV-positive squamous cell carcinoma [29], but could be treated effectively by carbon ion beam therapy [30]. From this perspective, pelvic irradiation with carbon ions might work efficiently to eradicate burdens of tumor recurrence, such as in the present case, although post-CIRT systemic treatments might have positively affected the outcome.

A phase 1/2 trial on CIRT for uterine cervical cancer without brachytherapy demonstrated a 2-year local control rate of 71%, even when 74.4 Gy (RBE) was used [31]. Because further escalation of the carbon ion dose was considered difficult considering the tolerance dose for the intestinal tract, we chose to add IGABT in combination with CIRT in our case. In IGABT, treatment planning based on the in-room CT obtained at each session contributes to minimizing the dose delivered to the intestinal tract. The present case indicates the potential efficacy of CIRT in combination with IGABT as a definitive local therapy for MNA of the uterine cervix, warranting further validation in a larger patient group.

The patient described in our case developed a series of multiple lung metastases over 70 months. The lung is recognized as an organ that frequently shows metastasis from MNA of the uterine cervix; a recently published multi-institutional study in 24 patients with MNA showed that half of the cases (12/24) were associated with recurrences, most commonly to distant sites ([75%] 9/12), frequently to the lungs ([56%] 5/9) [32]. This suggests the importance of treating lung metastases as part of the cure for MNA. In the literature, most MNA metastases were treated with chemotherapy, resulting in short-term relapse. By contrast, we were able to control metastatic lesions by SBRT, while ensuring the safety of the treatment in collaboration with the hospital specializing in stereotactic irradiation, leading to the patient surviving for 8 years with a high quality of life. Thus, the role of SBRT in combination with systemic therapy for metastatic MNA should also be further evaluated.

In summary, we report the first case of MNA of the cervix treated with CIRT in combination with brachytherapy. CIRT (55.2 Gy in 16 fractions) and IGABT achieved pelvic control for 8 years with acceptable adverse effects. After primary treatment, a series of multiple lung metastases, occurring over 70 months, were controlled by SBRT and systemic treatment. This case indicates the potential of a combination of CIRT and IGABT as a local treatment to eradicate MNA, which is a rare disease entity without a current standardized treatment.

## Data Availability

The data generated and analyzed in the current study are not publicly available due to the personal patient data included; however, they may be available from the corresponding author upon reasonable request.

## Abbreviations

CIRT: Carbon ion radiotherapy
CT: Computed tomography
EBRT: External beam radiotherapy
HR-CTV: High-risk clinical target volume
IGABT: Image-guided adaptive brachytherapy
MNA: Mesonephric adenocarcinoma
MRI: Magnetic resonance imaging
RBE: Relative biological effectiveness
SBRT: Stereotactic body radiotherapy
